# Supplementary material for: Chromothripsis during telomere crisis is independent of NHEJ, and consistent with a replicative origin
Source: Genome Res. 2019 May;29(5):737–49. doi: 10.1101/gr.240705.118 (PMC6499312; doi:10.1101/gr.240705.118)
Supplement: Supplemental Material [file supp_gr.240705.118_Supplemental_file_1.zip › contigs/annotated_contigs/DB115/contig.4.DB115_length_1358_mean_cov_11.7503681885.docx]

**DB115_length_1358_mean_cov_11.7503681885**

```
TACAACATTTGCGATTCATGGATTCATAAACAAGACCTAATAACAGGAGTTTTAACATAAATAGTAATGGAACTATTAGGGGAAAGGAA
 >chr15:47787457-47787751 - E=1e-164 p=1e-02
GGAAATTTACCTCTGAACTACATGCAATAGCTAACCCCTGCTACTCCACAACCTCAGTTTAAAAAGTAAGTTCTTTGGCATCAGTACAA

AGTTGAATCAGCTCCATGAGTCTAGATTTACAATAAGGCATAACATAAGCTTTAAGGGAGAATAGAACTTATTAACAAATGGAAAATCA

GAAAAGTTTTCTTTCTTTTCTAAAAA|A|TATATATATATCTCCATCCCACTCTCCAGATTTTTAAATGTACTTTAGAGGTTAGAAGAA
                           >chr15:47786709-47786950 - E=8e-133 p=2e-02
AAATAAGAGAAAAGAATTCAAGTATTTTGAATTATAATAAAATATGCTGCCAAATCCTTCAGCAACTGTTCATCATGCTAAATACATTA

TAATAACTATTCACTGGTCATATATATTCAAAATTCCCAAAATTATATATTAACAAATTATGTTCAAGATGCATAAAACTTAATGGG|A

AAA|TATAGCAAAGGAAAGCTACATTCCTTCTCTGAATACACAGTTGCACAAATTATTATTATTTATAGAGTTTCTTTAATCATGCACA
>chr15:47786987-47787457 - E=5e-259
ACCATAAGAACGACTTCCAGTTTTTTCTTTTCCTATTCAAAGCAGATTTTTAACAAGAAAATGGTGAGGCATCAGAAACTTAGTCCCTC

ATGGAGTTGACCTTTTAAATATTCAGTATTTTTATTGACTGTCCATTGTATTTTCTTCCCTTGAGATACTGGTATGGTAATCAAAGCGT

GGGAATAGTTAAACATTACTACAAAACCCCAAAGCCAAATGCATCCTCCACAAAAAACTGCATTTCTCTTCCAACAACTAACTGTGTTT

GTGATTAAATGCAAATATAAATGCTAGCAATCAAAACAATGGCTTGCAATCATGTGCAGAATGATTCATGAATTAGCGTGTGTGTGTGT

GTGTGTGTGTGTGTGTATCTGGAGAT|A|CAATGCCCAGCATTGGTAACTGCTTCAGATATATTGTAAAGTGCCTTTCTAGCTAAGATA
                            >chr15:47785402-47785692 - E=3e-162
TTGTGATAGAGCAAGTTTTCAATTCTAAAGAGAAGCCTCCACATTGACCCTGATGATGCTTTTACTTGCCAACAAAAAGACCACAAAAT

GAAAAGACTGAGAGGTGAGAGTTGCTAACAAAATTTCACATAGTGTGTTTCTCCACAGAGGTGTGTTTCCAAGGGCCACAGTTTTCCCT

ATGTGAATATCAAACTCCAAGAATGGGCTGTTGTTCACCTGTGCCTTATGCT|CACCTCCCCCCCACTCCTGTTACAGTACCCCAACCA

CCCCGGATCAGAACCCCCTCTCGGCCCCCC
```
